# When Fat Talks: How Adipose-Derived Extracellular Vesicles Fuel Breast Cancer

**DOI:** 10.3390/ijms26199666

**Published:** 2025-10-03

**Authors:** Maria Pia Cavaleri, Tommaso Pusceddu, Lucia Sileo, Luna Ardondi, Ilaria Vitali, Ilenia Pia Cappucci, Laura Basile, Giuseppe Pezzotti, Francesco Fiorica, Letizia Ferroni, Barbara Zavan

**Affiliations:** 1 Translational Medicine Department, University of Ferrara, 44121 Ferrara, Italy; 2 Maria Cecilia Hospital, GVM Care and Research, 48033 Cotignola, Italy; 3 Biomedical Engineering Center, Kansai Medical University, Hirakata 573-1191, Japan; pezzotti.giu@kmu.ac.jp; 4 Department of Clinical Oncology, Section of Radiation Oncology and Nuclear Medicine, 37122 Verona, Italy; francesco.fiorica@aulss9.veneto.it

**Keywords:** extracellular vesicles, adipocyte, breast cancer cells, exosome

## Abstract

Adipose tissue plays a crucial role in the tumor microenvironment (TME), where its secreted extracellular vesicles (EVs) are involved in the complex signaling between tumor cells and surrounding stromal components. This study aims to unravel the mechanisms through which adipocyte-derived EVs influence breast cancer (BC) progression. Human mesenchymal stem cells (hMSCs) were differentiated into adipocytes following a 21-day induction protocol that led to significant accumulation of lipid droplets within the cells. EVs were isolated from the conditioned medium of both hMSC-derived adipocytes and BC cells. Particle size distribution, morphology, and uptake into the recipient cell were investigated via nanoparticle tracking analysis, transmission electron microscopy, and fluorescence microscopy, respectively. Our results show that BC-derived EVs notably impaired cell viability and modulated the expression of key genes involved in apoptosis resistance within stromal cells. On the other hand, stromal-derived EVs significantly altered tumor cell behavior, indicating a dynamic, bidirectional exchange of bioactive signals. These findings underscore the pivotal role of EV-mediated communication in the tumor-stroma interplay, suggesting that adipocyte-cancer cell EV crosstalk contributes to the remodeling of the TME, potentially facilitating tumor progression.

## 1. Introduction

Breast cancer (BC) remains the most frequently diagnosed malignancy in women and constitutes a leading cause of global cancer mortality. While significant progress has been made in stratifying the disease through molecular subtyping and in tailoring therapeutic approaches accordingly, the persistent rise in incidence, particularly in younger and premenopausal women, underscores the critical influence of environmental and systemic factors beyond intrinsic tumor biology [1]. Among these, nutritional status, adipose tissue function, and metabolic inflammation have emerged as central, yet underappreciated, determinants of BC initiation, progression, and therapeutic response. The breast is anatomically embedded in a rich matrix of white adipose tissue, which serves not only as a structural element but also as an active endocrine and immunometabolic organ. Adipocytes within the mammary microenvironment engage in constant bidirectional communication with epithelial and stromal cells, secreting a diverse range of bioactive mediators including adipokines (e.g., leptin, adiponectin, resistin), inflammatory cytokines, steroid hormones, and lipids that influence proliferation, angiogenesis, immune cell recruitment, and epithelial–mesenchymal plasticity [2,3]. The oncogenic potential of this adipose–tumor axis is amplified in states of metabolic dysregulation such as obesity, in which adipose expansion and low-grade chronic inflammation create a fertile niche for malignant transformation [4].

Recent findings suggest that this crosstalk is not limited to soluble factors, but also occurs via extracellular vesicles (EVs). These nano-sized membrane-bound carriers of RNA, proteins, and metabolites have emerged as pivotal vectors of intercellular communication within the BC microenvironment. Both adipocytes and cancer cells release EVs that traffic molecular information across cellular boundaries, modulating gene expression, metabolic states, and immune phenotypes in recipient cells [5,6]. Adipocyte-derived EVs (AdEVs) can deliver pro-tumorigenic signals, such as miRNAs that suppress apoptosis or promote stemness, as well as metabolic substrates that fuel mitochondrial respiration and redox adaptation in cancer cells [7]. Conversely, tumor-derived EVs (TdEVs) reprogram local adipocytes into catabolic, inflammatory phenotypes, referred to as cancer-associated adipocytes, that in turn support tumor growth and dissemination [8]. The functional interplay between diet, adipose tissue activity, and vesicle-mediated signaling is beginning to reshape our understanding of tumor-stroma dynamics. Dietary patterns influence the qualitative and quantitative composition of circulating and tissue-specific EVs, modulating the immunometabolic tone of the host and altering the systemic inflammatory set-point. Polyphenol-rich diets, caloric restriction, and fasting-mimicking protocols have been shown to modify EV content in both adipose tissue and blood, potentially exerting indirect anti-tumor effects via reprogramming of the tumor microenvironment (TME) [9]. Thus, EVs may serve as molecular transducers of dietary signals, linking nutritional status to gene expression in tumors and their surrounding stromal elements. Taken together, these insights reveal a complex and dynamic tripartite dialogue between adipose tissue, tumor cells, and diet, orchestrated in part by EVs. This communication axis is not only essential for maintaining tumor-promoting homeostasis but also represents a promising target for preventive and therapeutic interventions. Elucidating the mechanisms by which diet-modulated AdEVs influence BC behavior holds the potential to unlock novel strategies for metabolic reprogramming of the TME.

In light of the growing evidence that adipose tissue plays an active role in shaping BC behavior through EV-mediated signaling, and that nutritional status may further modulate this dialogue, we sought to dissect the molecular underpinnings of this tripartite interaction. Specifically, we hypothesized that AdEVs not only modulate tumor cell phenotype and inflammatory responses but also actively contribute to the remodeling of the extracellular matrix (ECM), a key regulator of cell behavior within the TME.

The ECM is increasingly recognized as a dynamic and instructive component of solid tumors. Far from being a passive scaffold, the matrix provides biochemical and biomechanical cues that influence tumor cell adhesion, polarity, motility, immune infiltration, and response to therapy. Alterations in ECM composition, organization, and stiffness have been strongly associated with invasive progression, immune exclusion, and resistance to apoptosis in BC [10]. Components such as fibronectin, collagen I/III, tenascin-C, and matrix metalloproteinases are frequently dysregulated in the BC stroma, contributing to a pro-tumorigenic mechanical and signaling landscape [11]. Importantly, adipocytes can act as local architects of the matrix, secreting ECM components and matrix-remodeling enzymes. This activity may be amplified or altered through the vesicular transfer of regulatory molecules. To explore these hypotheses in a physiologically relevant setting, we established an in vitro model to study directional, paracrine, and vesicle-mediated communication under controlled conditions.

AdEVs have recently emerged in the literature as active regulators of BC progression, challenging the long-held view of adipocytes as passive bystanders in the TME. Several studies have demonstrated that EVs released from adipocytes or adipose tissue explants can transfer metabolic enzymes, lipids, and non-coding RNAs to BC cells, thereby promoting proliferation, mitochondrial activation, and remodeling of ECM pathways. Notably, work on obesity-associated adipose tissue has highlighted the ability of AdEVs to potentiate oncogenic signaling, including PI3K/AKT/mTOR and the epithelial-to-mesenchymal transition (EMT)-related cascades, thus contributing to a more aggressive tumor phenotype. While these studies have been instrumental in defining the role of AdEVs in tumor–stroma crosstalk, they have relied almost exclusively on two-dimensional cell cultures or ex vivo tissue explants, experimental systems that cannot fully capture the structural, spatial, and biomechanical complexity of tumor growth in vivo [12,13,14].

What has been missing to date is a rigorous evaluation of AdEV effects in three-dimensional organoid models, which more faithfully reproduce the cellular heterogeneity, polarity, and matrix interactions of native breast tumors. By integrating organoid assays into the study of AdEV–tumor communication, our work provides an essential advance beyond existing reports. We show that AdEVs not only reprogram cancer cell transcriptional networks toward EMT and apoptosis resistance, as previously described, but also drive a measurable expansion of organoid size, establishing a direct link between EV-mediated molecular changes and macroscopic tumor-like growth in a 3D context. This organoid-based evidence represents a significant conceptual and methodological advance, bridging the gap between simplified monolayer models and in vivo tumor biology, and offering a more physiologically relevant platform to interrogate the pro-tumorigenic potential of adipose-derived vesicles.

Within this system, we investigated whether AdEVs modulate the invasive behavior and transcriptional plasticity of tumor cells, with particular emphasis on genes involved in ECM production, remodeling, and mechanosensing. Conversely, we assessed how TdEVs influence adipocyte function, phenotype, and secretome composition, potentially reinforcing a feedback loop that promotes stromal activation and tissue stiffness. Our approach integrates gene expression profiling, high-resolution imaging, ECM composition analysis, and functional assays to interrogate the molecular circuits governed by EVs in the adipose–tumor–matrix axis. We posit that AdEVs act not merely as metabolic shuttles or inflammatory mediators, but also as modulators of tissue topology and mechanical integrity, ultimately influencing the ecological fitness of BC cells. By delineating the contribution of AdEVs to ECM remodeling and microenvironmental conditioning, this study provides new insights into how diet-responsive stromal components may orchestrate the physical and biochemical niche that enables BC progression and opens avenues for therapeutic strategies targeting the matrix-regulatory functions of EVs.

## 2. Results

### 2.1. Adipose Tissue Reconstruction

**Figure 1:**
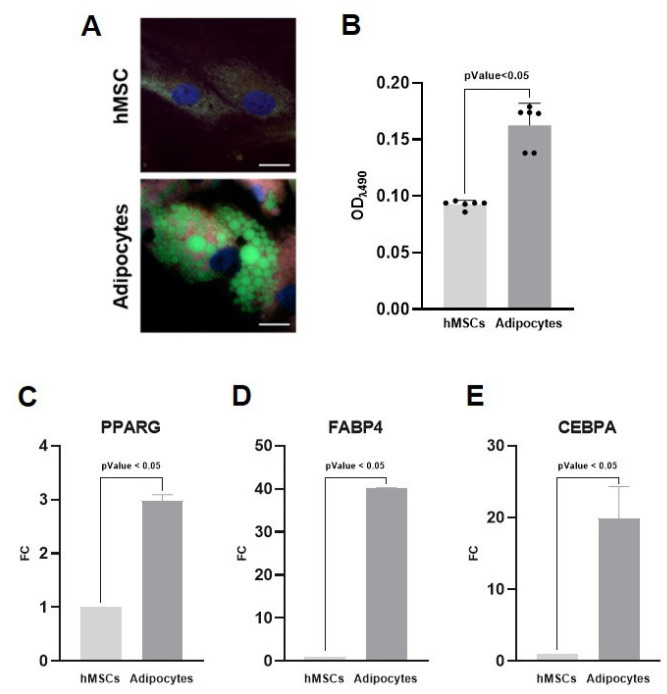
Creation of a model for harvesting adipocyte-derived extracellular vesicles (AdEVs). Human mesenchymal stem cells (hMSCs) were induced to adipocytes over 21 days. (**A**) BioTracker 488 staining shows differentiated adipocytes with prominent intracellular lipid vacuoles (green), which are absent in undifferentiated control cells. Nuclei are counterstained in blue. Scale bar 5 μm. (**B**) Quantification of intracellular lipid accumulation by Oil Red O staining demonstrated a marked increase in lipid content in differentiated adipocytes compared to controls. (**C**–**E**) Gene expression analysis of the key adipogenic marker genes *PPARG*, *FABP4*, and *CEBPA* showed significant upregulation in differentiated adipocytes compared to undifferentiated hMSCs cultured in basal medium. Sample size 9.

Human mesenchymal stem cells (hMSCs) were induced to undergo adipogenic differentiation over a 21-day period, which included an initial induction phase followed by a maintenance phase (refer to the Methods section for details). After 3 weeks, the acquisition of the adipogenic phenotype was assessed through lipid accumulation staining and quantification, as well as analysis of gene expression profiles. hMSCs maintained in basal growth medium served as undifferentiated controls (Figure 1).

The formation and intracellular distribution of lipid droplets, a hallmark of adipocyte differentiation, were visualized using BioTracker 488 staining (Figure 1A). hMSCs cultured under adipogenic conditions exhibited prominent cytoplasmic lipid vacuoles, whereas control hMSCs maintained in standard medium lacked detectable lipid structures, thereby confirming successful morphological differentiation. Furthermore, the intracellular lipid accumulation was quantified using Oil Red O staining (Figure 1B). The optical density (OD) of stained lipids was markedly increased in differentiated cells (0.163 a.u.) compared to control cells (0.093 a.u.), with statistical significance (*p* < 0.05). In addition, quantitative real-time PCR analysis of the key adipogenic marker genes peroxisome proliferator-activated receptor gamma (*PPARG*), fatty acid-binding protein 4 (*FABP4*), and CCAAT/enhancer-binding protein alpha (*CEBPA*) confirmed the differentiation into adipocytes (Figure 1C–E). These genes were significantly upregulated in hMSCs cultured under adipogenic conditions compared to undifferentiated controls, indicating robust activation of the adipogenic transcriptional program. Collectively, these results confirm effective adipogenic differentiation, establishing a suitable model for harvesting AdEVs.

### 2.2. Extracellular Vesicles Isolation and Characterization

**Figure 2:**
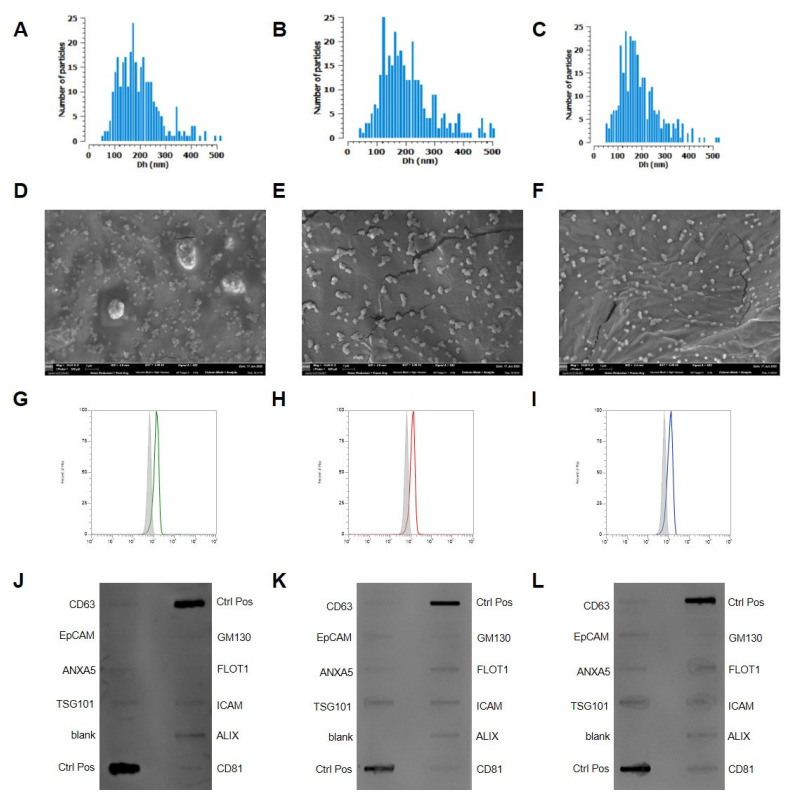
Characterization of adipocyte-derived extracellular vesicles (AdEVs) and tumor-derived extracellular vesicles (TdEVs). (**A**–**C**) Quantification and size distribution using the Corning VideoDrop system of AdEVs and TdEVs isolated from metastatic and non-metastatic cells, respectively. The *x*-axis represents mean diameter in nanometers (nm), while the *y*-axis indicates vesicle concentration expressed as particles per milliliter. (**D**–**F**) Transmission electron microscopy (TEM) images of AdEVs, and TdEVs from metastatic and non-metastatic cells, respectively. Scale bar = 100 nm. (**G**–**I**) Cytofluorimetric analyses of CD81 in AdEVs, TdEVs from metastatic and non-metastatic cells, respectively. (**J**–**L**) Western blot analyses of EV markers in AdEVs (**J**), TdEVs from metastatic (**K**) and non-metastatic cells (**L**), respectively. Sample size 9.

EVs were isolated from adipocyte and from BC cell cultures (metastatic and non-metastatic cell lines) via ultrafiltration. All cell types were maintained separately in EV-depleted medium for 24 h before conditioned medium collection. The conditioned media were subsequently clarified and subjected to ultrafiltration, and the retained EV fractions (AdEVs and TdEVs) were stored in aliquots for downstream characterization and cell treatment assays. AdEVs and TdEVs were characterized for particle size distribution and concentration, morphological features, and their internalization into recipient cells, as presented in Figure 2. Particle dimension analysis (Figure 2A–C) revealed that AdEVs displayed a size distribution ranging from 108 to 338 nm, with a mean diameter of 206 ± 102 nm, a modal peak at 175 nm, and a particle concentration of 3.01 × 10^9^ particles/mL. In comparison, TdEVs derived from metastatic cell line (MDA-MB-231) exhibited diameters between 115 and 354 nm, with a mean size of 219 ± 112 nm, a mode of 125 nm, and a concentration of 2.27 × 10^9^ particles/mL. The concentration of EVs derived from non-metastatic cell line (MCF-7) was determined to be 3.60 × 10^9^ particles/mL. Nanoparticle size distribution analysis demonstrated a heterogeneous population with diameters spanning from 106 to 315 nm, yielding an average size of 197 ± 96 nm and a predominant modal peak at 135 nm.

Transmission electron microscopy (TEM) further confirmed the nanoscale dimensions and typical vesicular morphology of all the isolated EVs (Figure 2D–F). The images revealed round to biconcave structures consistent with the size measurements obtained via VideoDrop (Figure 2A–C), supporting their classification as EVs. The classification and molecular profiling of EVs were carried out in strict adherence to the MISEV guidelines, ensuring compliance with internationally accepted standards for EV characterization. Flow cytometric analyses consistently demonstrated the robust expression of the canonical tetraspanin marker CD81 across all EV populations, thereby validating their vesicular nature (Figure 2G–I). These findings were corroborated by Western blot analysis, which further confirmed CD81 positivity, reinforcing the reliability and reproducibility of the classification pipeline. Beyond CD81, Western blot profiling revealed the presence of additional vesicle-associated proteins, including CD63, EpCAM, and Annexin, each of which is widely recognized as a defining component of EV subtypes (Figure 2J–L). The concurrent detection of these markers across orthogonal platforms not only substantiates the identity of the isolated vesicles but also underscores the methodological rigor and molecular integrity of the preparations. This multi-parameter validation strategy provides a solid framework for downstream functional studies and strengthens the translational relevance of the isolated EVs.

### 2.3. Bidirectional Communication Mediated by AdEV and TdEV

**Figure 3:**
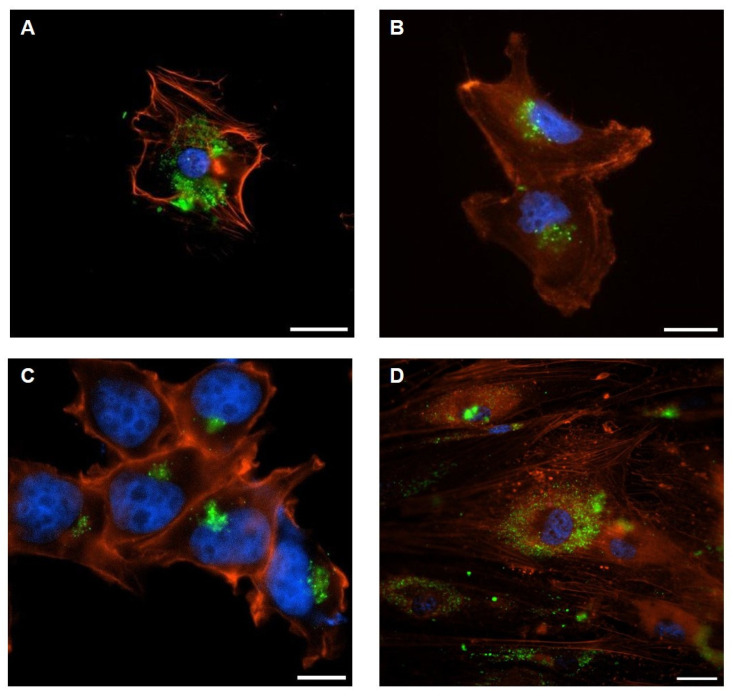
Representative confocal images of EV-internalization: (**A**) adipocytes incubated with TdEVs from metastatic cells; (**B**) metastatic cells incubated with AdEVs; (**C**) non-metastatic cells incubated with AdEVs; (**D**) adipocytes incubated with TdEVs from non-metastatic cells. Vesicles labeled with PKH67 (green), cell nuclei with DAPI (blue), and actin filaments with AlexaFluor555-Phalloidin (red). Scale bar = 10 µm.

To rigorously evaluate the dynamics of vesicle uptake, EVs were fluorescently labeled with the green lipophilic dye PKH67 and subsequently incubated with recipient cells under standardized conditions (Figure 3A–D). Following internalization, confocal laser scanning microscopy revealed discrete punctate fluorescent signals distributed throughout the cytoplasmic compartment, frequently accumulating in perinuclear domains demarcated by DAPI counterstaining (blue). The actin cytoskeleton, visualized through phalloidin-based red staining, provided an essential structural framework, thereby enabling the accurate spatial mapping of vesicle trajectories and intracellular localization. This multimodal labeling strategy confirmed that EVs were not merely attached to the plasma membrane but were actively trafficked into the intracellular milieu.

Detailed confocal analyses demonstrated that AdEVs were efficiently internalized by both metastatic BC cells (Figure 3A) and their non-metastatic counterparts (Figure 3C). Conversely, TdEVs released from metastatic BC cells (Figure 3B) as well as from non-metastatic lines (Figure 3D) exhibited robust uptake into differentiated adipocytes. The reciprocal nature of these interactions highlights a bidirectional vesicle exchange mechanism between adipose and tumor cell populations, consistent with a model in which EVs function as active vectors of intercellular communication across distinct cellular compartments.

Taken together, these findings provide compelling experimental evidence that both AdEVs and TdEVs engage in efficient, targeted, and bidirectional trafficking. The observed perinuclear accumulation strongly suggests endocytic uptake pathways leading to potential fusion with endosomal or lysosomal compartments, in agreement with current models of EV-mediated signaling. Beyond technical validation, these results substantiate the hypothesis that vesicle-mediated crosstalk between adipocytes and tumor cells constitutes a fundamental mechanism by which the TME and surrounding stromal tissues exchange regulatory cues. Such bidirectional vesicle internalization is likely to orchestrate functional reprogramming events, thereby contributing to tumor progression, metastatic potential, and the remodeling of the adipose niche.

The biological impact of EV-mediated communication was further dissected through gene expression profiling of recipient cells following prolonged exposure to vesicles. Metastatic BC cells and adipocytes were incubated for up to three days with 10^8^ particles/mL of AdEVs or TdEVs, respectively, while PBS-treated cells served as negative controls. This approach enabled us to capture sustained transcriptional reprogramming events that reflect the functional integration of vesicular cargo into recipient cell biology.

**Figure 4:**
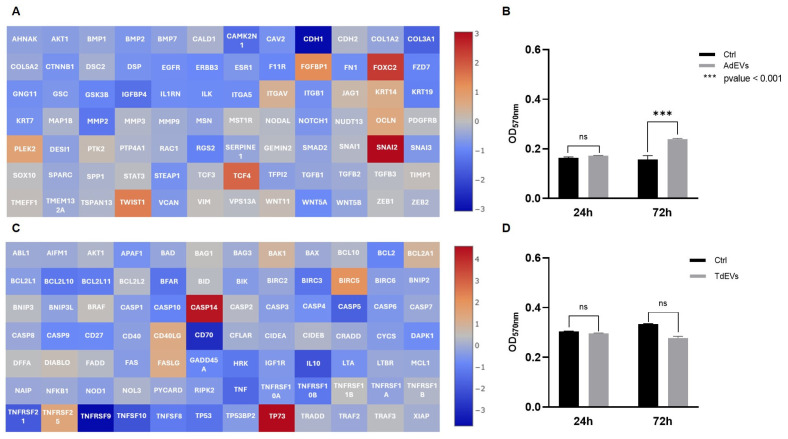
Communication between adipocytes and metastatic breast cancer (BC) cells is mediated by extracellular vesicles (EVs). (**A**,**C**) Gene expression profile of EV-treated cells: (**A**) Activation of epithelial-to-mesenchymal transition (EMT) program in metatastic BC cells after adipocyte-derived EV (AdEV) exposure; (**C**) modulation of apoptotic pathway genes in adipocytes treated with tumor-derived EV (TdEV) from metatastic BC cells. Heat map Log(2) fold change. (**B**,**D**) Cell proliferation after EV treatment over a 72 h period: (**B**) Proliferation of metastatic BC cells after AdEV treatment; (**D**) adipocyte proliferation following treatment withTdEV from metatastic BC cells. Sample size 9.

In metastatic BC cells treated with AdEVs, the transcriptional landscape strongly pointed to the activation of the EMT program (Figure 4A). This interpretation arises from the simultaneous upregulation of archetypal EMT regulators, including *CDH2*, *TWIST1*, *WNT11*, *VIM, SNAI2*, and *FOXC2*, and the suppression of genes responsible for maintaining epithelial integrity, such as *CDH1*, *COL1A2*, *COL3A1*, *COL5A2*, *KRT7*, *KRT19*, and *TFPI2*. The EMT framework is a well-defined cellular program through which epithelial cells progressively lose junctional stability and polarity, while acquiring mesenchymal features that favor motility, invasiveness, and metastatic dissemination. Thus, the observed gene expression profile is not a random set of changes, but a highly coherent shift that matches the canonical EMT signature. Functionally, this transcriptional reprogramming was reflected in a significant increase in metstatic BC cell proliferation (*p* < 0.001), as assessed by MTT assay (Figure 4B), thereby providing phenotypic confirmation that AdEVs promote a more aggressive, proliferative, and potentially invasive tumor phenotype.

Adipocytes exposed to TdEVs, by contrast, underwent a profound remodeling of apoptotic gene networks (Figure 4C). Here, the transcriptional balance shifted decisively toward survival: anti-apoptotic factors such as *BCL2A1*, *BCL2L2*, *BIRC5*, *XIAP*, and *TP73* were strongly upregulated, while pro-apoptotic mediators, including *BCL2L11*, *BAX*, *CASP3*, *CASP5*, *CASP8*, and *TP53*, were markedly repressed. The logic of this gene expression pattern is clear: TdEVs dampen the execution of programmed cell death while reinforcing cell survival pathways, effectively rendering adipocytes resistant to apoptotic stress. Importantly, this reprogramming does not simply extend adipocyte lifespan, it positions them as metabolically active and apoptosis-resistant stromal elements capable of supporting tumor growth. Interestingly, proliferation of TdEV-treated adipocytes displayed a slight but statistically nonsignificant reduction (Figure 4D), underscoring that the dominant biological effect of TdEVs lies not in driving proliferation but in stabilizing a pro-survival state.

Taken together, these findings illustrate how EVs function as powerful mediators of reciprocal transcriptional reprogramming within the adipose–tumor axis. AdEVs induce EMT-driven plasticity in BC cells, shifting them toward mesenchymal-like states with enhanced proliferative and invasive potential. In parallel, TdEVs rewire adipocyte gene networks to suppress apoptosis, creating a stromal microenvironment that is resilient, protective, and permissive to tumor expansion. This dual reprogramming establishes a bidirectional communication loop whereby vesicles from tumor and stromal compartments mutually reinforce tumor progression and microenvironmental adaptation.

Mechanistically, these transcriptional signatures align with well-established oncogenic signaling pathways. The EMT program observed in AdEV-treated BC cells is consistent with the activation of TGF-β and Wnt/β-catenin signaling cascades, both of which are canonical drivers of mesenchymal plasticity and metastatic competence. In parallel, the apoptosis-resistant profile induced by TdEVs in adipocytes maps onto survival-promoting pathways such as PI3K/AKT and NF-κB, which converge to suppress pro-apoptotic gene networks while enhancing anti-apoptotic effectors. These associations suggest that EV cargo not only triggers broad gene expression changes but likely engages specific intracellular signaling circuits, thereby providing a mechanistic framework that links vesicle-mediated communication to tumor progression and stromal adaptation.

**Figure 5:**
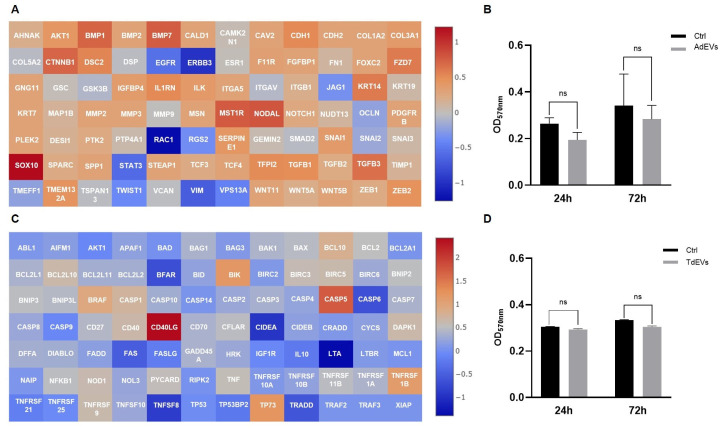
Communication between adipocytes and non-metastic breast cancer (BC) cells is mediated by extracellular vesicles (EVs). (**A**,**C**) Gene expression profile of EV-treated cells: (**A**) Activation of epithelial-to-mesenchymal transition (EMT) program in non-metastic BC cells after adipocyte-derived EV (AdEV) exposure; (**C**) modulation of apoptotic pathway genes in adipocytes treated with TdEV from non-metastatic BC cells. Heat map Log(2) fold change. (**B**,**D**) Cell proliferation after EV treatment over a 72 h period: (**B**) Proliferation of BC cells after AdEV treatment; (**D**) adipocyte proliferation following treatment with TdEV from non-metastatic BC cells. Sample size 9.

To further dissect the impact of AdEVs on non-metastatic BC biology, MCF-7 cells were incubated with 10^8^ particles/mL of AdEVs for up to 72 h, with PBS-treated cells serving as controls (Figure 5). Gene expression profiling uncovered a survival-oriented reprogramming of apoptotic gene networks (Figure 5A,C). Core anti-apoptotic modules were consistently upregulated, including *BCL2*, *BCL2A1*, *BCL2L10*, the NF-κB adaptor *BCL10*, and inhibitor-of-apoptosis genes *BIRC3* and *BIRC5*. Upregulation of *BRAF* and the *CD40/CD40LG* signaling dyad, along with increased *TP73*, further reinforced this pro-survival signature. Conversely, the extrinsic and executioner apoptosis pathways were attenuated, as indicated by the downregulation of *FAS/FASLG*, *CASP6*, and *TNFRSF21/TNFRSF25*. Collectively, these changes suggest that AdEVs endow non-metastatic BC cells with enhanced resistance to apoptotic stress, establishing a survival-competent transcriptional state. In parallel, AdEV-treated MCF-7 cells exhibited transcriptional activation of an EMT/ECM-remodeling axis. Growth factors and morphogenetic regulators such as *TGFB1/2/3*, *BMP1/2*, *NODAL*, and *NOTCH* were induced, together with transcriptional drivers of mesenchymal identity, including *SNAI1*, *FOXC2*, and *ZEB1/2*, as well as the matrix component *FN1*. These changes indicate initiation of mesenchymal traits and ECM reorganization. However, several canonical EMT and motility-associated drivers—including *TWIST1*, *STAT3*, *RAC1*, *RGS2*, and receptor tyrosine kinases *EGFR* and *ERBB3*—were simultaneously downregulated. This mixed expression profile argues against a full EMT conversion, instead supporting a state of partial or “hybrid epithelial/mesenchymal (E/M)” plasticity. Taken together, these data reveal that AdEVs impose a transcriptional configuration in non-metastatic BC cells that couples apoptosis resistance with early mesenchymal bias. On one hand, the upregulation of *BCL2*-family members, NF-κB adaptors, and inhibitor-of-apoptosis genes establishes a strong anti-apoptotic backbone, enhancing stress tolerance and cellular fitness. On the other hand, the induction of *FOXC2*, *ZEB1/2*, and *SNAI1* primes an EMT-permissive state characterized by ECM remodeling and phenotypic flexibility, without triggering the full migratory and invasive program that would require *TWIST1*, *STAT3*, and *RAC1*. This “hybrid E/M” state is mechanistically consistent with the non-metastatic background of MCF-7 cells: it enhances survival capacity and fosters conditions favorable to tumor persistence and expansion, yet restrains complete EMT activation and metastatic commitment. Mechanistically, these transcriptional signatures can be integrated into established oncogenic signaling frameworks. The survival bias is aligned with activation of NF-κB and PI3K/AKT signaling cascades, known to stabilize BCL2-family proteins and inhibitor-of-apoptosis genes. The EMT priming, meanwhile, reflects partial engagement of the TGF-β and Notch pathways, which drive ECM remodeling and mesenchymal transcription factors, but appears uncoupled from the full STAT3- and EGFR/ERBB3-dependent motility axis. Thus, AdEVs push non-metastatic BC cells into a survival-competent, EMT-primed hybrid state that may represent an adaptive intermediate, balancing growth advantage with restrained metastatic potential. In contrast to their metastatic counterparts, non-metastatic BC cells maintained in monolayer culture and treated with EVs failed to exhibit any appreciable proliferative advantage, as determined by MTT assay. Despite prolonged exposure to 10^8^ particles/mL EVs, the metabolic activity and viability indices of these non-metastatic cells remained comparable to PBS-treated controls, indicating that vesicle-mediated signaling in this context does not translate into a measurable increase in cell division rates. This observation underscores a fundamental divergence between metastatic and non-metastatic phenotypes, whereby only the former display a clear proliferative response to EV-driven reprogramming (Figure 5B–D).

**Figure 6:**
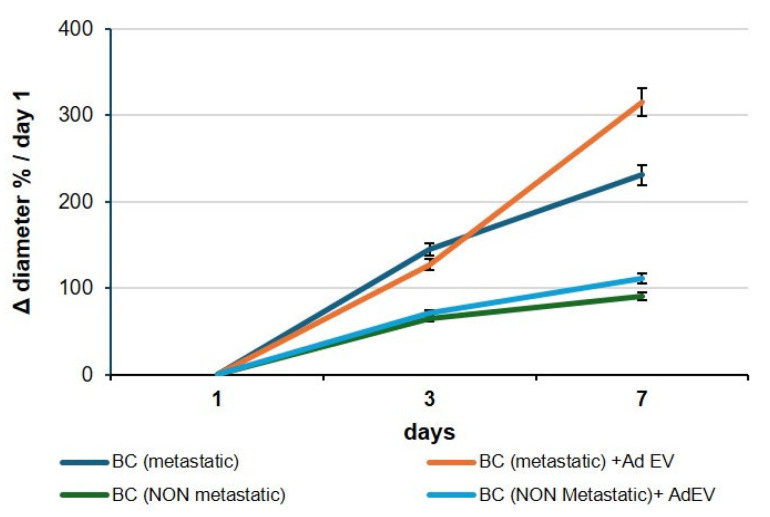
EV-induced expansion of breast cancer organoids. Representative images and quantification of breast cancer organoids cultured with 10^8^ particles/mL AdEVs for up to 72 h. Both metastatic (MDA-MB-231) and non-metastatic (MCF-7) organoids exhibited a significant increase in overall dimensions compared with PBS-treated controls. Data are presented as mean ± SEM; *p* < 0.05; *p* < 0.01. Sample size 9.

To quantify invasive behavior, BC organoids were embedded in a 3D fibrillar type I collagen matrix, a degradable and mechanically confining extracellular environment. In this context, an increase in the organoid’s projected diameter over time reflects radial advancement of the invasive front rather than simple volumetric growth. Collagen imposes steric and poroelastic resistance; outward expansion therefore requires cells at the periphery to generate actomyosin-driven protrusions, transmit traction through integrin–collagen adhesions, and remodel the matrix via pericellular proteolysis and fiber realignment, hallmarks of collective invasion. This biophysical coupling is the basis of the canonical spheroid invasion assay, in which the time-dependent increase in projected area (reported here as Δ diameter normalized to Day 1) is a quantitative surrogate of migration/invasion capacity. Consistently, proliferation alone is insufficient to drive boundary expansion under collagen confinement, whereas invasive behavior correlates with ECM remodeling and is known to be attenuated by MMP inhibition or increased matrix crosslinking. Accordingly, in our system, increases in organoid diameter within collagen are interpreted as enhanced invasion. The pro-metastatic consequences of EV exposure were rigorously assessed in three-dimensional BC organoid systems, which represent physiologically relevant models for capturing tumor growth kinetics (Figure 6). When cultured in the presence of 10^8^ particles/mL EVs, organoids displayed a marked and reproducible increase in overall dimensions compared with PBS-treated controls. Serial quantification of organoid diameters and volumetric expansion over a 72 h period demonstrated a statistically significant acceleration of growth rates, reflecting the sustained proliferative drive imparted by EV uptake. This dimensional enlargement, although a seemingly simple metric, constitutes a robust functional correlate of proliferative reprogramming, indicating that EVs act not merely as passive vesicular cargo carriers but as active drivers of tumor tissue expansion. Importantly, the increase in organoid size provides an experimental surrogate for tumor mass accumulation in vivo, thereby linking vesicle-mediated transcriptional changes to macroscopic growth behavior. Mechanistically, this proliferation-driven expansion is consistent with the molecular signatures detected in EV-treated cells, which revealed concurrent activation of anti-apoptotic modules and EMT/ECM-remodeling programs. The convergence of these pathways sustains cell survival while simultaneously priming cells for increased adaptability and resilience under growth stress, thus establishing conditions favorable to metastatic dissemination. From a translational standpoint, the dimensional increase in organoids in response to EVs can be interpreted as an early phenotypic manifestation of metastatic potential: larger organoid size reflects enhanced proliferative fitness, greater tissue plasticity, and the capacity to sustain growth despite environmental constraints. Collectively, these findings reinforce the concept that EV-mediated intercellular communication confers a survival- and proliferation-oriented state, which, even in non-metastatic contexts, drives tumor organoids toward a trajectory consistent with pro-metastatic progression.

## 3. Discussion

BC, particularly its invasive subtypes, remains one of the most prevalent and lethal malignancies worldwide, accounting for ~30% of newly diagnosed cancers and representing the leading cause of cancer-related mortality among women [15]. Mounting evidence highlights the pivotal role of the TME in driving disease progression, therapeutic resistance, and metastatic dissemination. Within this complex ecosystem, EVs have emerged as central mediators of intercellular communication, enabling the horizontal transfer of bioactive cargo that dynamically shapes tumor–stroma interactions.

In this study, we dissected the bidirectional molecular crosstalk mediated by EVs between BC cells and adipocyte-derived hMSCs, a stromal component increasingly recognized for its active contribution to tumor progression. AdEVs induced a robust EMT program in BC cells, characterized by the upregulation of master regulators (*TWIST1*, *FOXC2*, *CDH2*, *VIM*, *SNAI2*, and *WNT11*) and the concomitant downregulation of epithelial markers (*CDH1*, *KRT7*, *KRT19*) and ECM-related genes (*COL1A2*, *COL3A1*, *COL5A2*, *TFPI2*). These transcriptional shifts are indicative of an aggressive mesenchymal-like phenotype, reinforcing the concept that AdEVs potentiate tumor aggressiveness and stemness through EMT activation. Conversely, TdEVs significantly reprogrammed adipocyte transcriptomes, particularly in apoptotic pathways. Upregulation of anti-apoptotic genes (*BCL2A1*, *BCL2L2*, *BIRC5*, *XIAP*, *TP73*) coupled with suppression of pro-apoptotic effectors (*BCL2L11*, *BAX*, *CASP3*, *CASP5*, *CASP8*, *TP53*) conferred apoptosis resistance, suggesting that TdEVs promote the emergence of metabolically reprogrammed, inflammation-resistant adipocytes, which in turn sustain tumor growth. This reciprocal reprogramming underscores the non-passive, pro-tumorigenic role of adipocytes in the BC microenvironment. To further investigate the tumor-promoting potential of AdEVs, we analyzed their molecular cargo using the ExoCarta database (http://www.exocarta.org). AdEVs are enriched in proteins involved in ECM remodeling, metabolic adaptation, and signal transduction. Key ECM modulators include matrix metalloproteinase-3 (MMP-3), thrombospondin, and fibronectin. MMP-3 facilitates basement membrane degradation, thrombospondin contributes to ECM remodeling and angiogenic regulation [16], and fibronectin enhances adhesion, migration, and proliferation of recipient cancer cells [17].

Functional assays in non-metastatic BC cells (MCF-7) exposed to AdEVs (10^8^ particles/mL, up to 72 h) revealed a transcriptional reprogramming oriented toward apoptosis resistance. Upregulated genes included *BCL2*, *BCL2A1*, *BCL2L10*, *BCL10*, *BIRC3*, and *BIRC5*, alongside *BRAF*, *CD40/CD40LG*, and *TP73*, collectively creating a pro-survival signature. By contrast, *FAS/FASLG*, *CASP6*, *TNFRSF21*, *TNFRSF25* were consistently downregulated, reflecting suppression of death-inducing cascades. In parallel, EMT/ECM-remodeling drivers (*TGFB1/2/3*, *BMP1/2*, *NODAL*, *NOTCH*, *SNAI1*, *FOXC2*, *ZEB1/2*, *FN1*) were induced, while *TWIST1*, *STAT3*, *RAC1*, *RGS2*, *EGFR*, *ERBB3* were downregulated, consistent with a partial EMT program. This dual regulation suggests the emergence of a survival-competent, EMT-primed “hybrid E/M” state: non-metastatic BC cells acquire stress tolerance and mesenchymal bias (via *FOXC2*, *ZEB*, *SNAI1* and ECM remodeling), without fully committing to migratory or invasive behavior. Such EV-driven plasticity may represent a “pre-metastatic poise”, endowing tumor cells with growth competence and resilience while remaining non-invasive.

From a conceptual standpoint, this hybrid E/M state exemplifies tumor plasticity, where epithelial and mesenchymal features coexist to provide proliferative anchorage, stress resistance, and adaptability. These intermediate phenotypes are increasingly recognized as hallmarks of therapeutic tolerance, metabolic flexibility, and metastatic latency. Thus, AdEV-mediated reprogramming reinforces tumor cell survival and adaptability, contributing to disease persistence and therapeutic evasion.

At the metabolic level, AdEVs transport enzymes and lipid-binding proteins that fuel fatty acid oxidation and mitochondrial remodeling, including FABP4, Perilipin 1, and ceruloplasmin. FABP4 mediates lipid trafficking and metabolic coupling, Perilipin 1 regulates lipid droplet formation and lipolysis, while ceruloplasmin modulates redox balance [18,19]. AdEVs also deliver small GTPases such as RhoA and Rac1, potentially reshaping cytoskeletal dynamics and motility [20]. Lipidomic profiling highlights an abundance of phosphatidylcholine, ceramides, sphingomyelin, cholesterol, and sphingosine-1-phosphate (S1P). These bioactive lipids promote membrane fusion, metabolic activation, and inflammatory signaling. Ceramide- and cholesterol-rich domains facilitate vesicle uptake, while free fatty acids fuel β-oxidation, supporting energy production under nutrient stress. S1P and ceramides activate NF-κB and MAPK pathways, enhancing proliferation, survival, angiogenesis, and immune modulation [21,22].

Finally, AdEVs convey a diverse RNA repertoire—including coding mRNAs (PPARγ, C/EBPα), long non-coding RNAs, circRNAs (e.g., circ_0075932), and microRNAs (miR-155, miR-27a, miR-802-5p, miR-23a/b, miR-34a, miR-146a, miR-21, miR-29b). This RNA cargo orchestrates epigenetic reprogramming, apoptosis evasion, angiogenesis, immune modulation, and metabolic plasticity. For instance, miR-155 and miR-34a modulate macrophage polarization toward a pro-inflammatory M1 phenotype [23], while miR-23a/b and circ_0075932 suppress pro-apoptotic targets (TP53, CASP3), facilitating survival and chemoresistance [24]. Other EV-associated transcripts—including VEGF, DGAT2, PPARγ, and FABP4—support angiogenesis, lipid storage, and metabolic flexibility under stress [25]. miR-27a, miR-802-5p, and miR-21 modulate adipogenesis and tumor suppressor pathways, while miR-146a and miR-29b regulate inflammation and ECM remodeling [26]. Collectively, these RNA species establish a multifaceted reprogramming program that reinforces tumor aggressiveness, therapy resistance, and metastatic potential.

**Figure 7:**
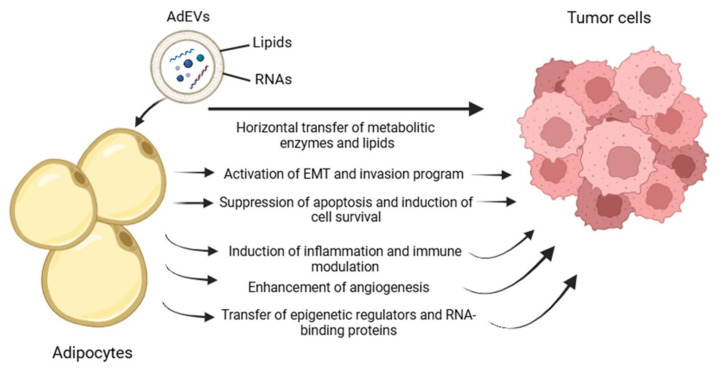
Schematic representation of the putative model of action by which adipocyte-derived extracellular vesicles contribute to tumor progression.

Taken together, our findings highlight multiple, convergent mechanisms through which AdEVs promote BC progression (Figure 7): (i) induction of EMT and invasion-associated programs, (ii) suppression of apoptosis and promotion of survival, (iii) metabolic and redox reprogramming, (iv) immune and inflammatory modulation, (v) angiogenic remodeling, and (vi) epigenetic and post-transcriptional regulation. This integrative view positions AdEVs as central orchestrators of tumor–stroma reciprocity and tumor plasticity, with profound implications for disease progression, metastasis, and therapeutic targeting.

## 4. Materials and Methods

### 4.1. Cell Cultures

hMSC (Lonza Bioscience, Basel, Switzerland) and BC cell line MDA-MB-231 and MCF-7 (Merck, Darmstadt, Germany) were cultured in Dulbecco’s Modified Eagle’s Medium High Glucose (DMEM-HG; EuroClone, Pero, Italy), supplemented with 1× penicillin-streptomycin (PS; Thermo Fisher Scientific, Waltham, MA, USA), 4 mM L-glutamine (L-GLU; EuroClone), and 10% fetal bovine serum (FBS; EuroClone, Pero, Italy). All cell lines were maintained at 37 °C in a humidified incubator with 5% CO_2_. Before EV isolation, the medium was replaced with EV-depleted culture medium consisting of DMEM-HG, 1× PS, 4 mM L-GLU, and 10% EV-depleted FBS (Thermo Fisher Scientific).

MDA-MB-231 (metastatic) and MCF-7 (non-metastatic) cells were seeded at a density of 5 × 10^5^ cells per T75 flask in complete medium until reaching approximately 70% confluence. Then, cell cultures were gently washed with pre-warmed PBS (37 °C), and EV-depleted culture medium was added. Cells were incubated for 24 h, and then the conditioned medium was collected for EV isolation.

hMSCs were seeded at a density of 5 × 10^5^ cells per T75 flask in complete growth medium until subconfluence. Adipogenic differentiation was induced over 21 days using a two-phase induction/maintenance protocol: first, the adipogenic induction medium consisting in DMEM-HG, 10% FBS, 1× PS, 0.01 mg/mL insulin, 1 µM dexamethasone, 0.2 mM 3-isobutyl-1-methylxanthine (IBMX), and 0.4 µM rosiglitazone (used in place of indomethacin); second, the adipogenic maintenance medium consisting in DMEM-HG, 10% FBS, 1× PS, and 0.01 mg/mL insulin. As previously described [27], the adipogenic differentiation was assessed by quantifying lipid accumulation before downstream applications (*i.e*., AdEV isolation and adipocyte treatment with TdEVs).

### 4.2. Lipid Quantification with Oil Red O Staining

In a 24-well plate, hMSCs previously subjected to adipogenic differentiation were stained with Oil Red O (Merck, Darmstadt, Germany) to quantify intracellular lipid accumulation [28]. Cells were washed with pre-warmed PBS (37 °C) and fixed with 4% paraformaldehyde for 20 min at room temperature (RT). After two PBS washes, cells were incubated with freshly prepared 0.35% Oil Red O solution in 60% isopropanol for 30 min. Excess dye was removed by washing with 60% isopropanol. The retained dye, associated with cellular lipids, was then extracted using 500 µL of absolute isopropanol with gentle agitation for 20 min at RT. The extracted dye solution was transferred to a 96-well plate, and absorbance was measured at 490 nm using a Perkin Elmer Victor3V Multilabel Plate Reader 1420 (Perkin Elmer, Waltham, MA, USA). Undifferentiated hMSCs were used as a negative control.

### 4.3. Isolation of Extracellular Vesicles

A total of 15 mL of conditioned medium was retrieved from BC cells and adipocyte cultures. EV isolation was performed by ultrafiltration technique using Amicon Ultra-15 centrifugal filters with Ultracel-100 regenerated cellulose membrane (Millipore, Burlington, MA, USA) [29]. Initially, the medium was centrifuged at 300× *g* for 10 min at 4 °C to remove cellular debris. The supernatant was transferred into the ultrafiltration device and centrifuged at 2000× *g* for 30 min at 4 °C. The retained cells were washed with PBS and subjected to a second centrifugation step at 2000× *g* for 30 min at 4 °C. The final retained EVs were resuspended in 1 mL of PBS. The enriched EV suspensions were aliquoted and stored at −20 °C until further use and designated as TdEVs and AdEVs.

### 4.4. Quantification and Size Characterization of Extracellular Vesicles

EV concentration and size distribution in TdEVs and AdEVs were evaluated using the Corning^®^ Videodrop (Corning Life Sciences, Durham, NC, USA). For each measurement, 8 µL of EV suspension was placed onto the sample slide. All measurements were performed in triplicate under optimized temperature and image saturation settings.

### 4.5. Transmission Electron Microscopy of Extracellular Vesicles

TEM imaging was employed to assess the morphology and size of TdEVs and AdEVs. Sample preparation and analysis were performed in collaboration with the Electron Microscopy Center of the University of Ferrara (Ferrara, Italy). EV samples (4 µL at a concentration of 10^8^ particles/mL) were deposited onto 200-mesh Cu/Pd grids, air-dried, and fixed with 2% paraformaldehyde and 1% glutaraldehyde in 100 mM PBS. Samples were stained with 2% uranyl acetate for 10 min, washed, and air-dried before observation using a Talos L120C G2 TEM (Thermo Fisher Scientific) [30].

### 4.6. Cell Treatments with Extracellular Vesicles

Adipocytes were treated with 10^8^ particles/mL TdEVs, and conversely, MDA-MB-231 cells were treated with 10^8^ particles/mL AdEVs. Cells were treated for 1 and 3 days for viability assays, and 24 h for fluorescence microscopy and RT-qPCR analyses. Untreated adipocytes and MDA-MB-231 cells served as negative controls.

### 4.7. Cell Viability Assay

Cell viability was assessed using the MTT assay kit (OZ BIOSCIENCE, Marseille, France) [31]. Adipocytes and MDA-MB-231 cells were seeded in 96-well plates at 5000 cells/well and treated, respectively, with TdEVs and AdEVs (10^8^ particles/mL) for 1 and 3 days. After treatment, cells were washed with 1× PBS and incubated with 100 µL of 1× MTT working solution [3-(4,5-dimethylthiazol-2-yl)-2,5-diphenyltetrazolium bromide] for 3 h at 37 °C. Formazan crystals were solubilized in 100 µL of solubilization solution, and absorbance was measured at 570 nm using a Perkin Elmer Victor3V Multilabel Plate Reader 1420.

### 4.8. Fluorescence Microscopy

To visualize lipid vacuoles in adipocytes derived from hMSCs, cells were seeded on glass coverslips (8000 cells/coverslip) and stained with BioTracker 488 (1 µg/mL, Merck). Cells were fixed with 4% PFA for 15 min, washed, incubated with 0.3 M glycine, and counterstained with DAPI using ProLong™ Gold Antifade Mountant (Invitrogen, Waltham, MA, USA).

EVs’ internalization was assessed by fluorescent labeling of TdEVs and AdEVs, with PKH67 (Sigma-Aldrich, Saint Louis, MO, USA), following an optimized nanoparticle labeling protocol [5]. EVs (20 mL) were ultracentrifuged at 100,000× *g* for 1 h at 4 °C (rotor Type 70.1 Ti, Beckman Coulter, Brea, CA, USA), resuspended in Diluent C, and labeled with 0.8 µL PKH67. Labeling was stopped with 10% BSA, and vesicles were washed, ultracentrifuged again, and resuspended in PBS. Target cells were incubated with labeled EVs (10^8^ particles/mL) for 12 h, fixed with 4% PFA, permeabilized, and counterstained with Alexa Fluor 555-conjugated phalloidin (Thermo Fisher Scientific). Imaging was performed with a Nikon Eclipse Ti-E Inverted LED Fluorescence Microscope equipped with a 60× oil immersion objective, ORCA-Flash4.0 V3 camera (Hamamatsu Photonics, Hamamatsu City, Japan), and pE-800 LED excitation source (CoolLED, Andover, UK).

### 4.9. RNA Extraction and Purification

Adipocytes treated with TdEVs and MDA-MB-231 cells treated with AdEVs (10^8^ particles/mL for 24 h) were lysed using 500 µL of TRIzol (Thermo Fisher Scientific) per well. After the addition of 100 µL of chloroform, lysates were incubated at RT and centrifuged at 12,000× *g* for 10 min at 4 °C. The aqueous phase was recovered and mixed with 250 µL isopropanol, incubated for 10 min, and centrifuged at 12,000× *g* for 15 min at 4 °C. RNA pellets were washed twice with 75% ethanol, centrifuged at 7500× *g* for 5 min, air-dried, and resuspended in 30 µL RNase/DNase-free water. RNA quality and quantity were assessed using a NanoDrop One spectrophotometer (Thermo Fisher Scientific), and samples were stored at −80 °C.

### 4.10. Organoid Production and Treatments

Three-dimensional cultures were carried out using Vitrogel Organoid 1 (The Well Bioscience, North Brunswick Township, NJ, USA) with the Cell Droplet method, following the manufacturer’s protocol. MDA-MB-231 and MCF-7 cells were detached from the culture flask by incubation with 0.25% trypsin-EDTA for 5 min. The enzyme was then diluted at a 5:1 ratio with complete medium containing 10% FBS. The cells were centrifuged at 200× *g* at room temperature for 5 min and resuspended in an appropriate volume of culture medium supplemented with hEGF. Cells were then counted and diluted to obtain 100 µL of suspension containing 3 × 10^5^ cells (3 × 10^6^ cells/mL). Organoids were exposed to AdEVs at 10^8^ mL^−1^ (NTA-based dosing) in 1–2% FBS assay medium. Controls included vehicle, heat-inactivated EVs (70 °C, 10 min), and RNase/Protease-treated EVs (cargo-dependency control). Media/EVs were renewed every 48 h. Each condition was tested in ≥3 independent biological replicates. Bright-field images were acquired at T_0_, day 3, 5, and 7 using fixed stage coordinates (≥1–3 fields/well). Organoid projected area (A, μm^2^) was segmented in Fiji/ImageJ (Fiji-win64) (automatic threshold with manual verification). The equivalent diameter was computed as

d = 2A/π.

Assuming sphericity, volume was estimated as

V = 43π(d/2)3

Primary endpoints were ΔA% and Δd% relative to T_0_; secondary endpoints included growth rate [(dt−d0)/t] and volume change. Outliers were flagged a priori (ROUT).

For each replicate, 6–8 organoids/condition were analyzed and averaged at the well level to avoid pseudoreplication. Data are mean ± SEM. Effects of dose and time were evaluated by two-way repeated-measures ANOVA with Greenhouse–Geisser correction and Benjamini–Hochberg FDR adjustment. Single-time comparisons used one-way ANOVA with Tukey’s post hoc test. Significance was set at α = 0.05; effect sizes (η^2^) and 95% CIs are reported where appropriate. Baseline size homogeneity: CV of T_0_ diameter < 15% per condition. Viability > 85% (paired organoids by AlamarBlue or Trypan Blue on sacrificial wells). No macroscopic core necrosis. EV dose stability verified by NTA (±10%) across the assay window.

### 4.11. Gene Expression Analysis

Adipogenic differentiation: RNA from adipocytes was reverse transcribed using SensiFAST cDNA Synthesis Kit (Meridian Bioscience, Newtown, OH, USA) according to the manufacturer’s instructions. qPCR was performed using SensiFAST SYBR No-ROX Master Mix (Meridian Bioscience) and primers (Thermo Fisher Scientific). Genes analyzed included PPARG, FABP4, and CEBPA, using B2M as a reference gene. Primer sequences are listed in Table 1. Amplification conditions: 95 °C for 2 min, followed by 40 cycles of 95 °C (5 s), 60 °C (10 s), and 70 °C (20 s). Melt curve analysis was performed from 72 °C to 95 °C.

Treated cells with TdEVs or AdEVs: cDNA was synthesized with RT^2^ First Strand Kit (Qiagen) and analyzed using RT^2^ SYBR Green ROX FAST Mastermix (Qiagen) and RT^2^ PCR Array (Qiagen) for Human Epithelial to Mesenchymal Transition or RT^2^ PCR Array for Human Apoptosis. Amplification: 95 °C for 10 min, followed by 40 cycles of 95 °C (5 s) and 60 °C (30 s). Reactions were run in triplicate using Rotor-Gene Q (Qiagen).

### 4.12. Statistical and Bioinformatics Analysis

Raw Ct values were analyzed using Q-Rex Software (version 2.0.0.39; Qiagen, Hilden, Germany), and statistical analyses were performed with GraphPad Prism 8 (GraphPad Software Inc., Boston, MA, USA) or with Excel (Microsoft Excel 2016). Gene expression was calculated using the 2^^-ΔΔCt^ method and expressed as Fold Change (FC). FC > 1 indicates gene upregulation; FC < 1 indicates downregulation relative to controls. Statistical significance (*p* < 0.05) was determined by appropriate tests, with significance thresholds denoted accordingly. In this study, the indication ‘sample size = 9′ refers to nine technical replicates, i.e., parallel wells/measurements performed under the same experimental condition within a single culture preparation.

## 5. Conclusions

This study provides compelling evidence that EV-mediated communication between BC cells and adipose-derived stromal cells plays a critical role in remodeling the TME toward a pro-tumorigenic state. This bidirectional exchange of regulatory signals, involving the activation of EMT programs in tumor cells and the suppression of apoptotic pathways in stromal adipocytes, underscores the transformative capacity of EVs in driving cancer progression beyond cell-autonomous mechanisms. Looking forward, several translational and mechanistic avenues merit exploration. First, targeting EV biogenesis and release, for instance, through pharmacological inhibition of neutral sphingomyelinase or components of the ESCRT machinery, may represent a novel approach to interrupt this malignant intercellular dialogue. Similarly, blocking EV uptake by recipient cells through the modulation of endocytic pathways or surface receptor interactions could attenuate the propagation of pro-tumoral phenotypes. Moreover, a deeper investigation into the molecular cargoes, including non-coding RNAs, proteins, and lipids selectively enriched within adipocyte- and tumor-derived EVs, could unveil new biomarkers of disease aggressiveness, therapeutic resistance, or microenvironmental conditioning. Elucidating the context-specific effects of exosomal signals on other stromal components, such as immune cells, fibroblasts, and endothelial cells, will further clarify the systemic impact of EVs in BC pathophysiology. Importantly, the selective engineering of EVs or EV-mimetic nanocarriers may also open new horizons for precision delivery of anti-cancer agents or reprogramming of the tumor stroma toward a suppressive phenotype. In this light, EVs stand not only as facilitators of disease progression but also as versatile platforms for diagnostic and therapeutic innovation. As research in the field of EVs advances, it becomes increasingly clear that integrating EV biology into the current framework of cancer treatment may offer critical leverage to overcome therapeutic resistance, prevent metastatic spread, and improve patient outcomes in BC and beyond.

## Figures and Tables

**Table 1:** Primer sequences used for gene expression analysis.

Gene	Forward Primer	Reverse Primer
*PPARG*	CAGGAGATCACAGAGTATGCCAA	TCCCTTGTCATGAAGCCTTGG
*FABP4*	TGACCTGGACTGAAGTTCGC	AAGCACAATGAATACATCATTACATCACC
*CEBPA*	GGACTTGGTGCGTCTAAGATGAG	GCATTGGAGCGGTGAGTTTG
*B2M*	CTGGTCTTTCTATCTCTTGTACTACACTG	CAAACCTCCATGATGCTGCTTA

*B2M*: Beta-2-Microglobulin; *CEBPA*, CCAAT: enhancer-binding protein alpha; *FABP4*: Fatty ac-id-binding protein 4; *PPARG*: Peroxisome proliferator-activated receptor gamma.

## Data Availability

Dataset available on request from the authors.

## Abbreviations

The following abbreviations are used in this manuscript:

AdEVs	adipocyte-derived EVs
BC	breast cancer
ECM	extracellular matrix
EMT	epithelial-to-mesenchymal transition
EVs	extracellular vesicles
hMSCs	human mesenchymal stem cells
TdEVs	tumor-derived EVs
TME	tumor microenvironment
